# Blended Vision Achieved by Combining High and Low Addition Power Diffractive Intraocular Lenses with Micromonovision: A Clinical Outcome

**DOI:** 10.1155/2020/6143832

**Published:** 2020-04-27

**Authors:** Masayuki Ouchi, Takuya Shiba

**Affiliations:** ^1^Masayuki Ouchi Eye Clinic, Kyoto, Japan; ^2^Department of Ophthalmology, Kyoto Prefectural University of Medicine, Kyoto, Japan; ^3^Roppongi Sgiba Eye Clinic, Tokyo, Japan

## Abstract

**Purpose:**

To evaluate the clinical outcome of blended vision combined with micromonovision (MMBV) using a +4 diopter (D) addition power (add) diffractive intraocular lens (MIOL) and a +2.75D add MIOL with a myopic target of −0.5 D.

**Methods:**

One hundred twenty eyes of 60 cases were enrolled. The +4 D add MIOLs were placed in the nondominant eye, the +2.75 D add MIOLs were placed in the dominant eye with a myopic target of −0.5 D in 30 cases (the MMBV group), and the +4 D add MIOLs were placed in both eyes in another 30 cases (controls). Postoperative clinical outcomes were compared between the two groups.

**Results:**

Compared with the controls, binocular uncorrected intermediate vision at 70 cm was significantly better in the MMBV group (*p*=0.02). Contrast sensitivity at 12 cycles per degree and the 6% and 12.5% low-contrast visual acuities were also significantly better in the MMBV group compared with the controls (*p* values = 0.05, 0.05, and 0.04, respectively). Uncorrected and corrected distance and near VA did not differ significantly between the two groups.

**Conclusion:**

MMBV provided a better intermediate VA, contrast sensitivity, and low-contrast VA than bilateral implantation of the +4 D add MIOL, while preserving comparable near and distance vision.

## 1. Introduction

Modern diffractive multifocal intraocular lenses (MIOLs) were first introduced with +4 diopter (D) near addition (add) power, and several studies have demonstrated that MIOLs provide reliable functional outcomes for distance and near vision. Subsequently, MIOLs with low add power (+3.00 or 3.25 D and +2.50 or 2.75 D) were developed. The +2.75 D add power at the IOL plane corresponds to +2.01 D at the corneal plane to improve intermediate vision at 50 cm.

Although a high add power MIOL can achieve excellent near visual acuity (VA) at reading distance, decreased contrast sensitivity is a well-known adverse effect of this type of IOL [1]. Conversely, in the low add power model, its fewer and wider concentric diffractive rings are thought to reduce the incidence and severity of unwanted postoperative complications, including deterioration of contrast sensitivity [2] and photic symptoms, and thus achieve a higher patient satisfaction rate [3]. Bilateral implantation of low add power multifocal IOLs, however, results in insufficient reading vision [4] because its near focal point is theoretically 50 cm.

Therefore, striking a balance between good near VA and contrast is challenging. To address this problem, we tried to achieve blended vision combined with micromonovision (MMBV). In this method, we inserted *a* + 2.75 D add power IOL in the dominant eye targeting −0.5 D myopia so that the near focal point can be shifted to approximately 40 cm while preserving the good contrast of this type of MIOL combined with *a* + 4 D add power MIOL in the nondominant eye. We investigated the clinical outcome of MMBV with careful evaluation of contrast sensitivity.

## 2. Patients and Methods

### 2.1. Patients and Intraocular Lenses

This study was carried out at a single institute, and ethics approval was obtained from the Institutional Review Board of Masayuki Ouchi Eye Clinic. Prior written informed consent for the use of medical records was obtained from all patients, and patient data were used in accordance with the tenets of the Declaration of Helsinki. This study included 120 eyes of 60 cataract patients who underwent bilateral MIOL implantation from March 2018 through June 2019. Exclusion criteria were patients with pathology of the cornea, macula, or optic nerve; severe opaque media other than cataract; history of ocular inflammation or surgery; corneal astigmatism of more than 1.5 D; and any difficulties with examinations, analyses, or follow-up.

### 2.2. Grouping

Sixty patients were divided into two groups. In the first group, a ZMB00 (ZMB: Abbott Medical Optics, Santa Ana, CA) MIOL was inserted into both eyes of 30 patients (the control group), and in the second group, both a ZMB and ZKB00 (ZKB: Abbott Medical Optics) were inserted in 1 eye each of 30 patients (the MMBV group).

### 2.3. Micromonovision

In the MMBV group, ocular dominance was assessed before surgery using the hole-in-the-card test. All patients underwent scheduled bilateral cataract surgery. The ZMB targeting emmetropia was first inserted in the nondominant eye, followed by the ZKB targeting −0.5 D myopia in the dominant eye. Dominant eyes were intended to have distance focus at 2 m and a near focal point at 40 cm, and nondominant eyes were intended to have a distance focus at infinity and a near focal point at 30 cm. In the usual monovision method, postoperative refraction is targeted for emmetropia in the dominant eye, and myopia in the nondominant eye. In contrast, in our method, postoperative refraction was targeted to be slightly myopic for the dominant eye and emmetropic for the nondominant eye. This strategy was based on the concept that higher contrast should be guaranteed with the higher contrast IOL in the dominant eye.

### 2.4. Intraocular Lens Power

The SRK/T formula was calculated, and the IOL constants were configured according to the manufacturer's calculation constants and the optimized values provided by the User Group for Laser Interference Biometry and Internal Optimization. In the control group, the IOL dioptric power was selected to target emmetropia in both eyes. In the MMBV group, the IOL dioptric power was selected to be −0.5 D in the dominant eyes, and in the nondominant eye, the IOL dioptric power was selected to target emmetropia, using the IOL power corresponding to the negative (myopic) predicted refractive outcome closest to zero or −0.5 D.

### 2.5. Surgical Procedures

All surgeries in both groups were performed by the same surgeon (M.O.) using topical anesthesia. After creating an initial side port incision, a cohesive viscosurgical device (sodium hyaluronate 1.0%: DisCoVisc1.0, Alcon Laboratories, Inc., Fort Worth, TX) was injected into the anterior chamber, and then a clear corneal incision was made in the superior quadrant (between 10 o'clock and 11 o'clock) using a 2.2-mm disposable metal blade. A continuous curvilinear capsulorrhexis was created, followed by hydrodissection and phacoemulsification of the nuclear fragments and aspiration of the cortex. After injecting a cohesive viscosurgical device (Healon, Abbott Medical Optics), a multifocal IOL, the ZMB, was implanted in both eyes in the control group. In the MMBV group, the ZMB was implanted in the nondominant eye and the ZKB was implanted in the dominant eye. In all eyes, the IOL was implanted in the capsular bag using an automated injector (AutoSert, Alcon Laboratories, Inc.) connected to a D-cartridge (Alcon Laboratories, Inc.). Irrigation and aspiration were performed to remove the ophthalmic viscoelastic surgical device, and all patients received an intracameral injection of 1 mg/0.1 mL moxifloxacin at the end of surgery.

### 2.6. Outcome Measures

Manifest spherical equivalent (SE), refractive sphere (RS), uncorrected and best-corrected distance VA (UDVA and CDVA), and corneal astigmatism (CA) were measured preoperatively and 3 months postoperatively.

Monocular and binocular UDVA and CDVA; uncorrected and best-corrected near VA (UNVA and CNVA); uncorrected and distance-corrected multidistance VA at 0.3, 0.4, 0.5, 0.7, and 1.0 m; and contrast sensitivity and low-contrast VA with 12.5% and 6% contrast were measured at 3 months postoperatively. The multidistance VA test was performed using a multidistance chart (TMI-V5; Precision Vision, La Salle, IL) with a 5-card set for near to intermediate testing with a cord attached to ensure an accurate testing distance. The contrast sensitivity test was performed with stimulus spatial frequencies between 3 and 18 cycles per degree (cpd) using a CSV-1000 (Vector Vision, Greenville, OH, USA) at 2.5 m under mesopic conditions, and low-contrast VA was measured with the system chart of a liquid crystal display that displays characters with different contrasts (SC-1600; NIDEK Co., Ltd., Gamagori, Japan).

Spectacle dependency for distance and near vision, and halo symptoms were assessed by patient questionnaire. Halo symptoms were also classified according to the patient's response as follows: severe, moderate, slight, and none.

### 2.7. Statistical Analysis

Statistical analyses were performed using R version 3.4.1 (the R Foundation: https://www.r-project.org/). The normality of the data distribution was examined using a histogram. Student's *t*-test was used to analyze patient age and preoperative CA, as both the control and MMBV group datasets were normally distributed and had equal variance. Welch's *t*-test was used to analyze preoperative SE and postoperative low-contrast VA because they did not have equal variance. Mann–Whitney's *U* test was used to analyze preoperative UDVA and CDVA; postoperative monocular and binocular UDVA, CDVA, UNVA, and CNVA; uncorrected and distance-corrected multidistance VA; and contrast sensitivity because both or one of them was not normally distributed. The Kruskal–Wallis test was used for multiple comparisons of monocular CA, and the Steel multiple comparison test was used for comparisons of monocular SE and RS because only the ZKB was inserted targeting −0.5 D. The results are presented as mean ± standard error of the mean. Categorical variables were compared using the chi-square test or Mann–Whitney's *U* test. Any differences with a *p* value of less than 0.05 were considered statistically significant.

### 2.8. Results

One hundred and twenty eyes of 60 patients (25 men and 35 women) were included in this study. Preoperative baseline data are shown in Table 1. Age, ratio of men and women, and the other continuous variables did not differ significantly between the two groups.

Table 2 shows the postoperative refractive data of control group eyes and the ZMB-implanted and ZKB-implanted eyes of in the MMBV group. Both SE and RS had a significantly myopic value in the ZKB-implanted eyes as they had a target refraction of −0.5 D. The groups did not differ significantly in postoperative corneal astigmatism.

### 2.9. Visual Acuity

Monocular VA and binocular VA are shown in Table 3. No significant difference in monocular UDVA was detected among three groups: control group eyes, ZMB-implanted eyes in the MMBV group, and ZKB-implanted eyes in the MMBV group, though only ZKB was implanted with a refractive target of −0.5 D (*p*=0.08). Also, monocular CDVA, UNVA, and CNVA were not significantly different among the three groups (*p*=0.34, 0.57, 0.92, respectively).

The values for binocular UDVA, CDVA, UNVA, and CNVA are shown in Table 3. None of the values differed significantly between the control and MMBV groups.

### 2.10. Multidistance Visual Acuity

**Figure 1:**
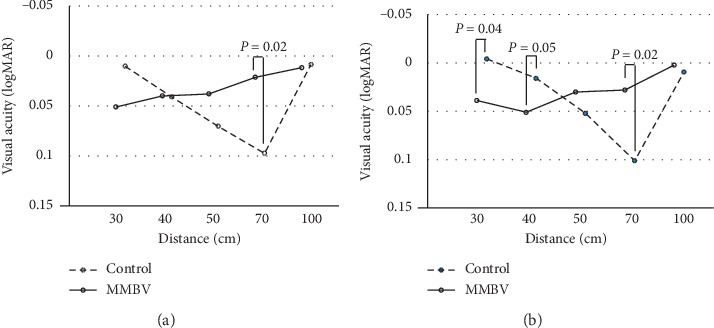
(a) Uncorrected binocular multidistance VA test. The MMBV group had significantly better intermediate VA at 70 cm than the control group (*p*=0.02, Mann–Whitney's *U* test) while no significant difference in near VA at 30 cm and 40 cm was detected. (b) Distance-corrected binocular multidistance VA test. The MMBV group had significantly worse near VA at 30 and 40 cm but better intermediate VA at 70 cm than the Control group (*p*=0.04, *p*=0.05, and *p*=0.02, respectively, Mann–Whitney's *U* test).

Uncorrected and distance-corrected binocular multidistance VA results are shown in Figure 1. Intermediate VA at 70 cm was significantly better in the MMBV group than in the control group (*p*=0.02), although no significant difference was detected between groups in near VA at 30 cm and 40 cm in the uncorrected multidistance VA test (Figure 1(a)). On the other hand, the MMBV group had significantly worse near VA at 30 and 40 cm (*p*=0.04 and 0.05, respectively) in distance corrected, i.e., without micromonovision conditioned, multidistance VA (Figure 1(b)). Distance-corrected intermediate VA at 70 cm, however, remained significantly better in the MMBV group than in the control group (*p*=0.02: Figure 1(b)). Uncorrected VA at 70 cm in all 60 subjects was distributed with a standard deviation of 0.10. The difference of means between the two groups was 0.078, and we can reject the null hypothesis that the means of the MMBV and control groups are equal with a probability (power) of 0.856 when the Type I error probability associated with this test of this null hypothesis is 0.05.

### 2.11. Contrast Sensitivity

**Figure 2:**
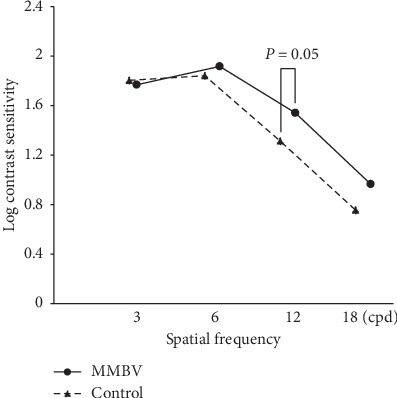
Binocular contrast sensitivity. The MMBV group had significantly better contrast sensitivity at 12 cpd than the control group (*p*=0.05, Mann–Whitney's *U* test).

The mean binocular log contrast sensitivity for high spatial frequency (12 cpd) was 1.31 ± 0.24 in the control group and 1.54 ± 0.34 in the MMBV group. The MMBV group had significantly better contrast sensitivity (*p*=0.05). Also, the MMBV group had better contrast sensitivity at 18 cpd (control, 0.76 ± 0.26; MMBV, 0.97 ± 0.21), but no significant difference could be detected because the contrast sensitivity at the highest spatial frequency could not be measured in some patients (Figure 2). The contrast sensitivity at 12 cpd of all 60 subjects in both groups was distributed with a standard deviation 0.288. The difference in the means between the two groups was 0.231, and the statistical power was calculated to be 0.875 when the Type I error probability associated with this test of this null hypothesis is 0.05.

### 2.12. Low-Contrast VA

**Figure 3:**
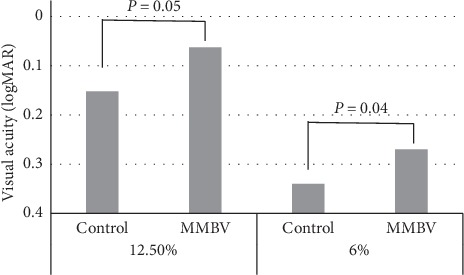
Binocular low-contrast VA with 12.5% contrast and 6.0% contrast. The MMBV group had significantly better VA at both 12.5% and 6.0% contrast than the control group (*p*=0.05 and *p*=0.04, respectively, Welch's *t*-test).

The results of the binocular low-contrast VA test with 12.5% and 6.0% contrast are shown in Figure 3. VA at 12.5% and 6% contrast was significantly better in the MMBV group compared with the control group. The contrast VA at 12.5% of all 60 subjects within both groups was distributed with standard deviation 0.12. The difference in the means between the two groups was 0.089, and the statistical power was calculated to be 0.806 when the Type I error probability associated with this test of this null hypothesis is 0.05.

### 2.13. Patient Questionnaire

Of the 30 patients, 1 patient in the control group occasionally used eyeglasses for distance vision, and all patients in the MMBV group were completely independent of spectacles; the difference between the groups was not statistically significant (*p*=0.32). For near vision, 5 patients in the control group occasionally needed eyeglasses, and in the MMBV group, 6 patients needed eyeglasses occasionally and 1 patient needed eyeglasses constantly (*p*=0.49). In the questionnaire about visual symptoms, 13 (43.3%) of the 30 patients in the control group reported having “halo or glare symptoms”. In the MMBV group, 15 of the 30 patients (50%) reported having visual symptoms. None of the patients in either group, however, marked the symptom as severe (*p*=0.86; Table 4).

### 2.14. Discussion

The findings of the present study demonstrated that the MMBV group, i.e., blended vision with ZKB implantation targeting −0.5 D combined with ZMB implantation targeting emmetropia, had significantly better bilateral uncorrected intermediate VA at 70 cm while preserving similar 30 cm and 40 cm UNVA and UDVA compared with the control group having bilateral ZMB implantation. This is likely due to the ZKB working better for intermediate vision, and only 0.5 D myopic targeting can enhance near focus. Targeting −0.5D is a trade-off between UNVA at 40 cm and distance VA greater than 2.0 m, although it did not have a great impact on bilateral UDVA in the MMBV group.

The contrast sensitivity test showed that high spatial frequency was significantly better in the MMBV group than in the control group, and the MMBV group also had significantly better low-contrast VA. This finding might be due to the relatively small amount of decrease in the contrast sensitivity provided by a low addition model. Further, these findings suggest that MMBV achieved clear vision bilaterally, and the ZKB lens preserved high contrast.

Ravikumar et al. [5] estimated the bifocal image of bilateral implantation of low add power diffractive IOLs combined with monovision and showed that the near edge of the focal depth expanded with the amount of added myopia. Our data are consistent with theirs in that the UNVA at 30 and 40 cm of the MMBV group was comparable to that in the control group. Moreover, based on the distance-corrected multidistance VA test (Figure 1(b)), if micromonovision was not combined, blend vision with ZMB and ZKB produced worse UNVA at 30 cm and 40 cm compared with the implantation of ZMB in both eyes.

On the other hand, decreased contrast sensitivity is well-known to be the most critical adverse event of diffractive IOLs, and waxy vision or hazy vision leads to postoperative patient dissatisfaction [6, 7] or explantation of diffractive IOLs [8]. Kamiya et al. reports that the most common reason for multifocal IOL explantation is decreased contrast sensitivity [8]. A retrospective review of dissatisfaction cases after multifocal IOL implantation revealed that the most common presenting symptom is blurred vision, and 90.8% [6] or 65% [9] of these cases were implanted with +4 D add MIOLs. In contrast, the present study demonstrated that diffractive multifocal IOLs with low add power not only significantly improve intermediate VA but also provide contrast VA similar to that of monofocal IOLs [10] and better contrast sensitivity than the higher add power models [2]. Also, our cases that underwent MMBV might benefit from this feature of the low add power model.

An extended range of focus IOL (EDOF) is one strategy used to address this issue, but EDOFs are reported to produce insufficient near VA [11], and micromonovision combined therapy was also reported [12]. The Concerto Study Group [11] reported that binocular UNVA was 0.12 (logMAR) in monovision combined EDOF IOL-implanted cases while in our case series, the binocular UNVA was 0.06, indicating that the ZMB might work well in our MMBV method than EDOF IOL implanted with a myopic target. The aim of the monovision method in the EDOF is to compensate for the insufficient near vision of bilateral EDOF implantation, but, on the other hand, the aim of the MMBV is to effectively use the higher contrast multifocal IOL to contribute to both intermediate and near vision.

Although this study is limited by the relatively small number of patients, power analysis indicated that the statistical power was sufficient for all of main outcomes.

In conclusion, MMBV, bended vision with +2.75 D near add power MIOL implantation targeting −0.5 D myopia combined with +4 D near add MIOL implantation targeting emmetropia, provides better contrast sensitivity and intermediate VA than bilateral implantation of +4 D near add power MIOLs while preserving comparable near and distance vision. Further studies are needed to investigate the optimal strategy for use of MIOLs with different add powers. The selection of MIOLs will likely continue to increase in the near future, and understanding the features of each IOL will allow the surgeon to customize MIOL options.

## Figures and Tables

**Table 1:** Patient characteristics and baseline data.

Parameter	Control	MMBV	
Mean age (y)	64.2 ± 9.2 (47–79)	68.7 ± 7.4 (46–78)	*p*=0.07^*∗∗*^
Sex (men/women)	13/17	12/18	*p*=0.5^§^
UDVA (logMAR)	0.63 ± 0.59 (−0.08–2.0)	0.64 ± 0.53 (−0.17–1.70)	*p*=0.72^∫^
CDVA (logMAR)	0.15 ± 0.23 (−0.08–2.0)	0.14 ± 0.23(−0.17–0.70)	*p*=0.60^∫^
SE (D)	−2.37 ± 7.00 (−14.1–3.25)	−1.93 ± 4.46 (−10.5–3.5)	*p*=0.10^†^
CA (D)	0.71 ± 0.48 (0–1.75)	0.61 ± 0.52 (0–1.25)	*p*=0.16^*∗∗*^
AL (mm)	25.38 ± 1.63 (21.69–27.01)	24.13 ± 1.71 (21.10–28.23)	*p*=0.23^∫^

UDVA, uncorrected distance visual acuity; CDVA, best-corrected distance visual acuity; logMAR: logarithm of the minimum angle of resolution; SE, manifest spherical equivalent; CA, corneal astigmatism; D, diopter. ^*∗∗*^Student's *t*-test, ^§^Fisher's exact probability test, ^∫^Mann–Whitney's *U* test, and ^†^Welch's *t*-test.

**Table 2:** Postoperative refractive data.

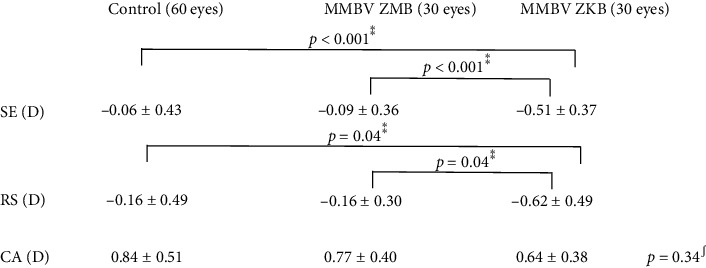

ZMB, ZMB00-implanted eye; ZKB, ZKB00-implanted eye; SE, manifest spherical equivalent; RS, refractive spherical equivalent; CA, corneal astigmatism. ^*∗∗*^Steel's multiple comparison test and ^∫^Kruskal–Wallis's test.

**Table 3:** Postoperative visual acuity.

Monocular	Control (60 eyes)	MMBV ZMB (30 eyes)	MMBV ZKB (30 eyes)	*p* value
UDVA	−0.05 ± 0.13	−0.01 ± 0.15	0.05 ± 0.23	*p*=0.08^*∗∗*^
CDVA	−0.17 ± 0.28	−0.10 ± 0.22	−0.12 ± 0.07	*p*=0.34^*∗∗*^
UNVA	0.17 ± 0.18	0.19 ± 0.20	0.22 ± 0.18	*p*=0.57^*∗∗*^
CNVA	0.04 ± 0.11	0.04 ± 0.10	0.04 ± 0.10	*p*=0.92^*∗∗*^
Binocular	Control (60 eyes)	MMBV (60 eyes)		
UDVA	−0.10 ± 0.07	−0.09 ± 0.11		*p*=0.72^∫^
CDVA	−0.12 ± 0.07	−0.15 ± 0.13		*p*=0.24^∫^
UNVA	0.02 ± 0.14	0.06 ± 0.13		*p*=0.45^∫^
CNVA	0.02 ± 0.12	0.03 ± 0.10		*p*=0.89^∫^

ZMB, ZMB00-implanted eye; ZKB, ZKB00-implanted eye; UDVA, uncorrected distance visual acuity; CDVA, best-corrected distance visual acuity; UNVA, uncorrected near visual acuity; CNVA, best-corrected near visual acuity. ^*∗∗*^Kruskal–Wallis's test and ^∫^Mann–Whitney's *U* test.

**Table 4:** Postoperative outcomes.

	Number (%)		*p* value (Mann–Whitney *U* test)
	Control	MMBV	
*Spectacle dependency (far)*			
Never	29 (96.7)	30 (100)	0.32
Occasional	1 (3.3)	0 (0)	
Always	0 (0)	0 (0)	

*Spectacle dependency (near)*			
Never	25 (83.3)	23 (76.7)	0.49
Occasional	5 (6.7)	6 (20.0)	
Always	0 (0)	1 (3.3)	

*Visual symptoms (halo or glare)*			
Severe	0 (0)	0 (0)	0.86
Moderate	6 (20)	4 (13.3)	
Slight	7 (23.3)	11 (36.7)	
None	17 (56.7)	15 (50)	

## Data Availability

The datasets generated and/or analyzed during the current study are available from the corresponding author upon reasonable request.

## Additional Points

*Highlights.* blended vision by combining monovision using +4 D and +2.75 D addition power MIOLs achieved better contrast and intermediate vision than bilateral +4 D addition power MIOLs, while preserving comparable near/distance vision.
